# ATF3 Positively Regulates Antibacterial Immunity by Modulating Macrophage Killing and Migration Functions

**DOI:** 10.3389/fimmu.2022.839502

**Published:** 2022-03-16

**Authors:** Yuzhang Du, Zhihui Ma, Juanjuan Zheng, Shu Huang, Xiaobao Yang, Yue Song, Danfeng Dong, Liyun Shi, Dakang Xu

**Affiliations:** ^1^ Department of Laboratory Medicine, Ruijin Hospital, Shanghai Jiao Tong University School of Medicine, Shanghai, China; ^2^ Department of Immunology and Medical Microbiology, Nanjing University of Chinese Medicine, Nanjing, China

**Keywords:** *Staphylococcus aureus*, activating transcription factor 3 (ATF3), antibacterial, macrophages, migration

## Abstract

The clinical severity of *Staphylococcus aureus* (*S. aureus*) respiratory infection correlates with antibacterial gene signature. *S. aureus* infection induces the expression of an antibacterial gene, as well as a central stress response gene, thus activating transcription factor 3 (ATF3). ATF3-deficient mice have attenuated protection against lethal *S. aureus* pneumonia and have a higher bacterial load. We tested the hypothesis that ATF3-related protection is based on the increased function of macrophages. Primary marrow-derived macrophages (BMDM) were used *in vitro* to determine the mechanism through which ATF3 alters the bacterial-killing ability. The expression of ATF3 correlated with the expression of antibacterial genes. Mechanistic studies showed that ATF3 upregulated antibacterial genes, while ATF3-deficient cells and lung tissues had a reduced level of antibacterial genes, which was accompanied by changes in the antibacterial process. We identified multiple ATF3 regulatory elements in the antibacterial gene promoters by chromatin immunoprecipitation analysis. In addition, Wild type (WT) mice had higher F4/80 macrophage migration in the lungs compared to ATF3-null mice, which may correlate with actin filament severing through ATF3-targeted actin-modifying protein gelsolin (GSN) for the macrophage cellular motility. Furthermore, ATF3 positively regulated inflammatory cytokines IL-6 and IL-12p40 might be able to contribute to the infection resolution. These data demonstrate a mechanism utilized by *S. aureus* to induce ATF3 to regulate antibacterial genes for antimicrobial processes within the cell, and to specifically regulate the actin cytoskeleton of F4/80 macrophages for their migration.

## Introduction

*Staphylococcus aureus* (*S. aureus*) is a major human pathogen that causes severe respiratory infections (1). Innate immune cells, mainly macrophages and neutrophils, act as gatekeepers in their interactions with *S. aureus*, killing the strain by phagocytosis to clear the infection (2). The important role of macrophages in protecting from *S. aureus* infection by using a wide range of killing mechanisms has attracted increasing attention. *S. aureus* has evolved multiple strategies to survive, such as manipulating and evading macrophages (2, 3). Macrophages and *S. aureus* affect the outcome of infection through various direct interactions. In addition, *S. aureus* has developed resistance to a variety of antibiotics; thus, effective treatment strategies for these bacteria are limited (4). *S. aureus* can exploit the immune system to evade the host’s defense system. Therefore, *S. aureus* is a serious threat to human health, and new treatment strategies are needed.

Although neutrophils are important antibacterial cells, their capability to produce inflammatory cytokines/chemokines is more limited than that of macrophages. Depletion of macrophages has been reported to significantly affect the host’s defense response and impede bacterial clearance, suggesting that control of infection is dependent on innate subpopulations of cells rather than on adaptive immune cells (5). In addition, adoptive transfer experiments have shown that macrophages primarily mediate the prevention of staphylococcal reinfection. Macrophages release large amounts of proinflammatory cytokines, chemokines, and antimicrobial peptides (AMPs), and by recruiting immune cells to coordinately fight against pathogens (6).

Macrophages sense and clear the invading microbial pathogens. *S. aureus* initial Toll-like receptor (TLR)-2 mediates local production of soluble mediators, including cytokines, chemokines, and AMPs (7). Such an inflammatory response can also promote the production of IL-17 to orchestrate the host immune response. Recent findings have suggested that IL-17 and IL-22/IL-23 regulate the function of macrophages, thereby inducing the expression of AMPs, such as regenerated islet-derived protein 3 (Reg3) and defensins, which can kill or inactivate microorganisms (8, 9). Therefore, inactivation of IL17, IL-22, and IL-23 leads to an increased bacterial load in the lungs, exacerbation of *S. aureus* pneumonia, and higher mortality (8, 10). Given the critical role of TLR2 and IL-17/IL-22/IL-23 pathways in host defense responses, it is important to elucidate the mechanisms involved and search for factors with regulatory potential.

Activating transcription factor 3 (ATF3) is a hub cellular stress response gene in the pathogenesis of the disease. For modulation of infection and inflammation, ATF3 encodes a member of the ATF/cyclic adenosine monophosphate (cAMP) response element (CRE) binding (CREB) transcription factor family (11). ATF3 plays an important role in the negative feedback loop response by suppressing TLR-mediated cytokine expression (12). This implies that ATF3 is a key regulator of host resistance to invasive pathogens and inflammatory diseases, including *S. aureus* infection (13). However, the exact mechanisms involved in ATF3-related regulation of proinflammatory cytokines, chemokines, and AMPs for the antimicrobial effects in *S. aureus* infection are still unclear. Furthermore, the role of ATF3 in *S. aureus* infection and the mechanism of its action remain to be determined.

In the present study, we demonstrated that ATF3 can promote bacterial clearance through the regulation of the host’s defense response and thus alleviate lethal *S. aureus* pneumonia. During *S. aureus* infection, the expression level of ATF3 closely correlated with the level of the host’s AMPs. We found that ATF3-deficient mice or cells had a higher bactericidal loading. Furthermore, we found that ATF3 positively correlated with AMPs gene expression (e.g., Reg3 that ATF3 transcription factors directly regulated AMPs genes *via* chromatin immunoprecipitation analysis of putative binding sites, consistent with macrophage bactericidal and migratory functions. ATF3 also affected the migration of F4/80 macrophages in the lungs, which correlated with actin filament severing through ATF3 regulation. Our data suggested that ATF3 plays an important role in early Staphylococcus aureus. infection through positive regulation of the host immunity against bacterial infection by regulating macrophage Reg3 expression, AMPs gene-mediated bacterial clearance, and F4/80 macrophage recruitment.

## Materials and Methods

### Mice

ATF3 -/- mice were reported by us previously (14). Sex- and age-matched (8–10 weeks) WT and ATF3 KO mice were used during the whole experiments. The mice were bred and kept on a 12 h reverse light/dark cycle and were provided with adequate water and food under specific pathogen-free conditions. The animals were transferred and housed in an animal facility for 3 d of stable housing prior to any experiments. The experiments were conducted in strict accordance with the National Institutes of Health Guide for the Care and Use of Laboratory Animals, approved by the Ethics Committee of Ruijin Hospital, Shanghai Jiao Tong University School of Medicine.

### Cell Culture

RAW 264.7 murine macrophages were maintained in Dulbecco’s modified Eagle’s medium (DMEM) containing 10% fetal calf serum at 37°C in a 5% CO_2_-humidified incubator. For bone marrow-derived macrophages (BMDMs), isolation and differentiation were performed in line with our published procedure with minor changes (15). Briefly, we flushed the tibias and femurs with precooled phosphate-buffered saline (PBS) using a 25-gauge needle and a 5 mL syringe. Then, the collected bone marrow was gently resuspended into single-cell suspension, and sometimes it was necessary to use red blood cell lysis solution (Sangon Biotech, Shanghai, China) to lyse the red blood cells in it. The cells were centrifuged at 700*g* for 4 min at room temperature. Then, they were resuspended using a 10 mL conditioned medium (DMEM + 10% fetal bovine serum (FBS) 1% penicillin/streptomycin, and 30% L929) and transferred into a 10-cm cell culture dish. Three days later, 10 mL of fresh conditioned medium was used to replace the old medium in the Petri dishes, and most of the cells were found to be stuck to the Petri dishes. After 7 d of cultivation, fresh medium was replaced, and the cells were used for experiments.

### *S. aureus* Growth and Labeling Conditions

The MRSA strain (USA300) reported by our previous study (16) was cultured in Luria Bertani broth at 37°C until its stable growth phase, and then collected by centrifugation at 8,000 rpm for 5 min. The bacteria were washed three times with PBS and resuspended to an optical density of 0.8 at 600 nm. Then, 500 µL of the bacterial suspension was incubated with an equal volume of 5.0 μM solution of 5(-and 6) carboxyfluorescein diacetate succinimidyl ester (CFDA/SE, Selleck) for 30 min in the dark at 37°C. Subsequently, the stained bacteria were washed three times with PBS and the resuspended bacteria were used for later experiments.

### Pneumonia Model

For lung bacterial infections, a total volume of 40 μL of PBS containing *S. aureus* (5 × 10^6^ CFU/mouse) was injected into the trachea of mice. For survival experiments, we used 2 × 10^8^ CFU/mouse of *S. aureus* and observed their survival 48 h after the infection. The survival rate of the mice was monitored every 3 h.

### Bronchoalveolar Lavage Fluid (BALF) Collection

BALF collection was performed in line with our published procedure with minor modifications (16). Briefly, we sacrificed the mice by cervical dislocation and exposed the trachea. A 20-gauge catheter was used, and 0.8 mL of PBS was dripped into the lungs and gathered into clean tubes. This process was repeated four times to collect about 3 mL of BALF from each mouse. We used a cell counting plate to count the total number of cells in the alveolar lavage fluid and a flow cytometer to count the number of macrophages and neutrophils in BALF. The remaining BALF was then centrifuged at 700 g for 5 min, and supernatants were stored at −80°C until cytokine analysis.

### Internalization and Killing of Bacteria

To evaluate the phagocytic capability, macrophages were incubated with CFSE-labeled *S. aureus* (MOI 10) for the indicated time periods. Then, the infected cells were washed using PBS, and extracellular bacteria were eliminated by treatment with lysostaphin (20 μg/mL) for 30 min. Macrophages were then collected, and intracellular bacterial loads were quantified by flow cytometry. To determine the bactericidal capability of macrophages, the cells were seeded on coverslips for 24 h and incubated with CFSE-labeled *S. aureus* (MOI 10) for 2 h. The cells were then washed and further cultured in a fresh medium containing lysostaphin (2 μg/mL) for the indicated time periods. Thereafter, the cells were fixed, and the nuclei were counterstained with DAPI, and then they were observed by fluorescence microscopy.

### Macrophage Killing Assay

The intracellular killing assay was conducted following a previously described procedure with slight modifications (17). BMDM cells from WT and ATF3 KO mice were infected with *S. aureus* (MOI 10) for 2 h. The cells were then washed and treated with a medium containing gentamicin (300 µg/mL) for 30 min to kill the extracellular bacteria. Then, the cells were further cultured in a fresh medium containing gentamicin (100 µg/mL) for the indicated time periods (2 h, 6 h, 12 h, and 18 h). Subsequently, the cells were washed several times with PBS and then lysed with 0.1% Triton X-100 to release the bacteria inside the cells. To estimate the number of bacteria, the lysate was serially diluted with sterile PBS, applied to LB plates, and incubated overnight in a dedicated bacterial incubator.

### Quantitative PCR

Total RNA was extracted using TRIzol reagent (Thermo Fisher Scientific, Waltham, MA, USA) in accordance with the manufacturer’s guidelines. One microgram of total RNA was reverse-transcribed to cDNA using SuperScript II (Invitrogen, Carlsbad, CA), and qRT-PCR was performed using SYBR Green technology (Takara, Tokyo, Japan). β-actin was used for normalization, and data were analyzed through the ΔΔCt method. The primers used for the detection in the study were synthesized by GENEWIZ (Suzhou, China), and sequences are shown in **Supplementary Table 1**.

### Myeloperoxidase (MPO) Activity Assay

MPO activity was assessed as an indicator of neutrophil accumulation in lung tissues by using Myeloperoxidase (MPO) Activity Fluorometric Assay Kit (#K745-100, BioVision, California, USA) in accordance with its instruction.

### Immunofluorescence Microscopy

WT and ATF3 KO BMDM cells were seeded on glass slides and stimulated with *S. aureus* for the indicated time. The cells were washed twice with prewarmed PBS and fixed with 4% paraformaldehyde solution for 15 min at room temperature. We washed the samples and permeabilized them in 0.1% Triton X-100 for 20 min. After washing the samples three times with PBS, we added the fluorescent phalloidin staining solution to each coverslip and incubated them in a covered container for 50 min at room temperature. We again washed the samples three times with PBS, and recorded immunofluorescence images by laser scanning confocal microscopy.

### Histological Analysis of Lung Tissues

For histological analysis, mouse lung samples were thoroughly washed in PBS, fixed in 4% (wt/vol) paraformaldehyde for 24 h, embedded in paraffin, and sliced into 5-μm-thick sections. Hematoxylin and eosin staining was performed in line with standard procedures. For immunostaining, the lung sections were deparaffinized, hydrated, and blocked in Dulbecco’s phosphate-buffered saline (DPBS)containing 2% normal goat serum. The slides were then stained with the indicated primary antibody and biotin-conjugated secondary antibody and then incubated with streptavidin-conjugated horseradish peroxidase (HRP). Finally, the slides were incubated with DAB reagent and counterstained with hematoxylin for observation.

### Cytokine Measurement

The levels of TNFα, IL-6, IL-1β, and IL-12 in the cell culture supernatants and BALF were measured by ELISA kits (R&D Systems, Minneapolis, MN) in accordance with the manufacturers’ guidelines.

### Transwell Migration Assay

BMDMs (1 × 10^5^ cells) were seeded onto the top chamber of an 8-μm pore transwell insert (Corning, NY, USA) in a 24-well plate with media containing 2% FBS. After 36 h, the media within the transwell inserts were carefully removed, and the cells were fixed with 2% paraformaldehyde and stained with 0.2% crystal violet. Cells that did not migrate across the transwell membrane were wiped from the top of the chamber. The migrated cells were viewed using a Nikon DS-F2 microscope. The inserts were then incubated with 30% acetic acid, and the absorbance was read at 570 nm. The experiment was repeated three times.

### Western Blot

The cell lysate was prepared with SDS-lysis buffer (Beyotime, Shanghai, China). Total protein concentration was measured by Nano-100 Micro-Spectrophotometer. Protein was then separated by 10% SDS-polyacrylamide microgel and transferred to 0.45-μm polyvinylidene difluoride membranes (Invitrogen). The PVDF membranes were then blocked in Tris-buffered saline containing 5% nonfat dry milk (w/v) (Sangon Biotech) in Tween-20 (TBST) for 1 h at room temperature. After that, the membranes were incubated with the indicated primary antibodies overnight. Then, they were incubated with a secondary antibody conjugated with HRP. We used Tanton™ Chemistar High-sig ECL Western Blotting Substrate (ECL) to display the signal. Full blots of images cropped for presentation are presented in **Supplementary Material**.

### Chromatin Immunoprecipitation Assay

The ChIP assay was performed using the ChIP-IT Express Magnetic Chromatin Immunoprecipitation kit (53008, Active Motif, Carlsbad, CA, USA) according to the manufacturer’s instructions. The chromatin solution was immunoprecipitated using either an anti-ATF3 antibody (CST, USA) or normal anti-IgG antibody (CST, USA) and then incubated with magnetic beads overnight at 4°C with rotation. Next, the DNA-Antibody complexes were washed sequentially by CHIP buffer1 and CHIP buffer2 and then eluted with elution buffer AM2. Then, RT-PCR was performed using purified DNA fragments and primers directed to the specific area spanning the putative ATF3-binding motif. Primer pairs used for ChIP are shown in **Supplementary Table 2**.

### Generation of Stable Cell Lines

The plasmids pLVX-flag-REG3β-IRES-Puro and pLVX-flag-REG3γ-IRES-Puro were purchesed from Shanghai Xitubio biotechnology Co., Ltd. To generate stable cell lines expressing REG3β or REG3γ, pLVX-flag-REG3β-IRES-Puro or pLVX-flag-REG3γ-IRES-Puro plasmid together with the packaging plasmids (psPAX2+pMD2.G) were transfected into human embryonic kidney 293T (HEK293T) cells using EZ Trans transfection resgents (Life iLab Bio-Technology, China). The supernatants containing the virus were harvested after 48 h post–transfection and filtered using a 0.45-μm filter. Cells were incubated with viral supernatants plus equal complete medium in the presence of 5 μg/mL polybrene (Sigma) for 24 h. After infection, positive clones were selected by puromycin selection and infection efficacy were validated by immunoblotting assays.

### Statistical Analyses

Unless otherwise stated, all data are expressed as the mean ± SD of three independent experiments. Student’s *t* test or one-way analysis of variance was used for comparison between groups. *P* values < 0.05 were considered statistically significant. Kaplan–Meier survival analysis with log-rank test was used to evaluate the survival curve. All calculations were performed using the Prism 8 software program (GraphPad Software).

## Results

### ATF3 Plays a Host Protective Role in Pneumonia Caused by *S. aureus*

**Figure 1:**
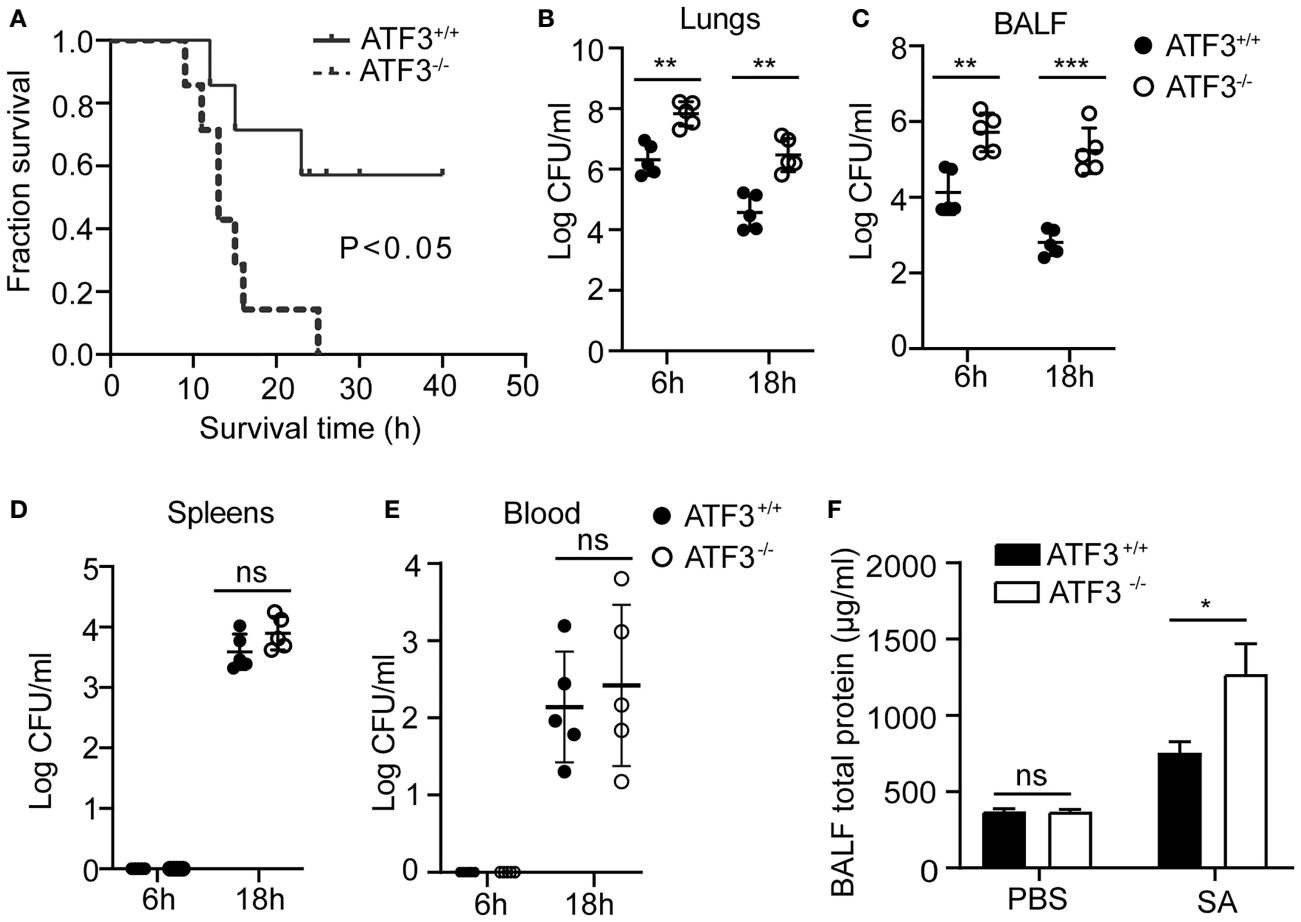
ATF3 provides protection against *S. aureus* infections. **(A)** WT and ATF3 KO mice were challenged intratracheally with 2 × 10^8^ CFU/mouse of a lethal inoculum of *S. aureus* (USA 300) and then observed to survive for 48 h. For the functional analysis, WT and ATF3 KO mice were infected intratracheally with a sublethal *S. aureus* inoculum (5 × 10^7^ CFU/mouse) and then euthanized at 6h and 18hafter infection to quantify the bacterial load in the lungs **(B)**, BALF **(C)**, spleens **(D)**, and blood **(E)**. **(F)** Total protein in BALF was measured. ns, *P* > 0.05, *P < 0.05, **P < 0.01, ***P < 0.001.

To explore the potential role of ATF3 in the host’s lung defense against *S. aureus* infection, we used a lethal dose of *S. aureus* (US 300) (2 × 10^8^ CFUs/mouse) to infect WT and ATF3 KO mice intratracheally and observed survival patterns for 48 h. Although all ATF3 KO mice died within 24 h, 60% of WT mice survived more than two days after infection (**Figure 1A**). To determine whether the difference in survival was due to the difference in bacterial load in various organs, we measured the bacterial load in the lungs, BALF, and extrapulmonary organs after infecting mice with a sublethal inoculum (5 × 10^7^ CFU) of *S. aureus*. Compared with WT mice, ATF3 KO mice had a higher bacterial load in the lungs and BALF, while there was no significant difference in spleen and blood bacterial load at 6 and 18 h after infection (**Figures 1B**–**E**). Meanwhile, the total protein (a measure of lung leakage) in the BALF of ATF3 KO mice was higher than that in the WT mice (**Figure 1F**). This suggests that ATF3 has a protective function against lethal *S. aureus* pneumonia and limits the host bacterial load.

### ATF3 Promotes Macrophage Recruitment and Enhances Resistance to *S. aureus*

**Figure 2:**
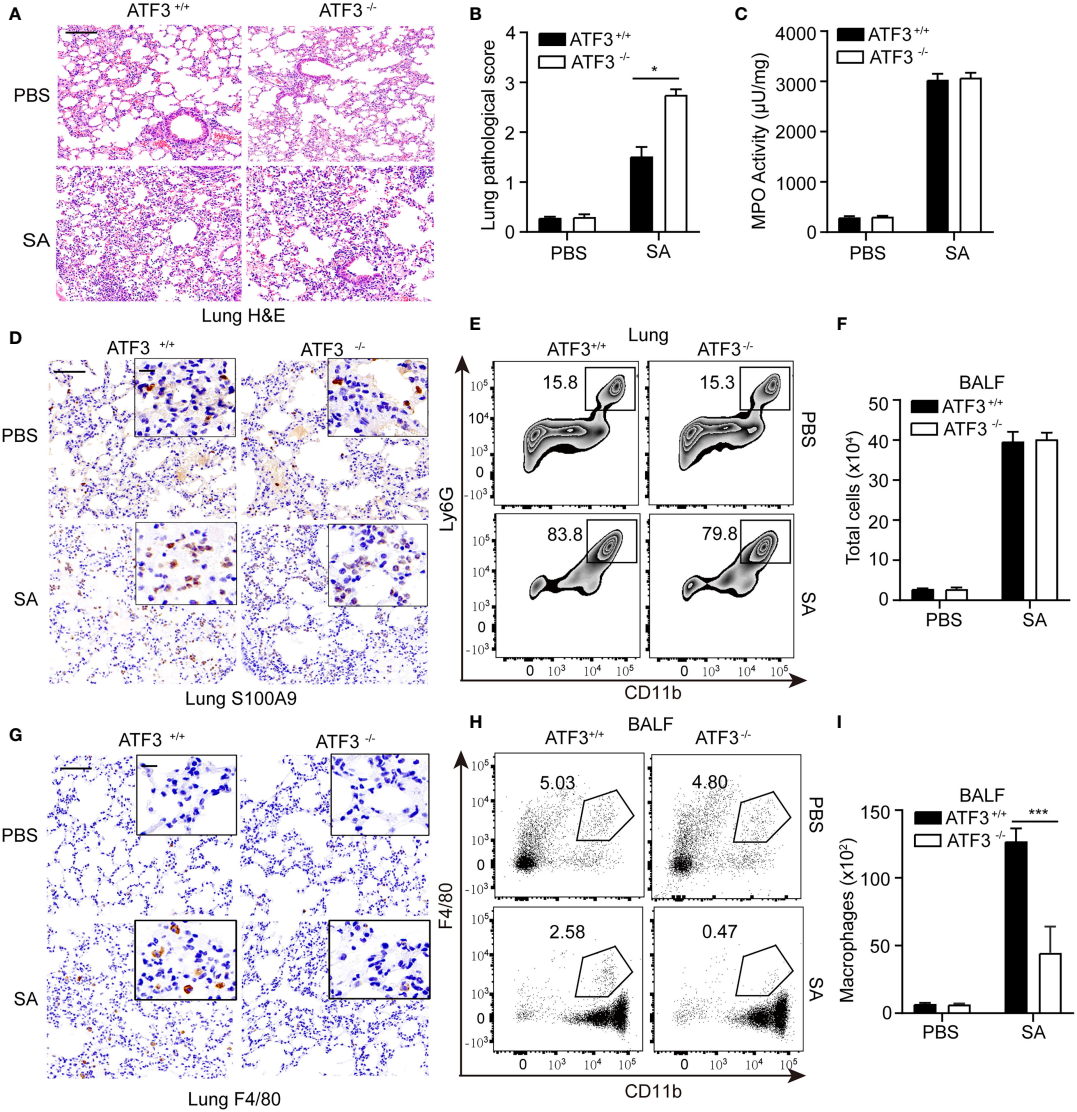
Macrophages confer host protection in WT mice compared with the ATF3 KO. **(A)** Tissue sections of lung tissues stained with hematoxylin and eosin. **(B)** Pathological damage scores of lung tissues. **(C)** Measures of MPO activity as quantified by ELISA in lung extracts from WT or ATF3 KO mouse after S. aureus infection. **(D)** Representative images of WT and ATF3 KO mice lung tissues were assessed for neutrophils (S100A9) after S. aureus infection. **(E)** Representative zebra plot showing CD11b+ Ly6G+ cells from WT or ATF3 KO mice lung tissues after S. aureus infection. **(F)** Mice BALF was collected; counts of total cells are shown. WT and ATF3 KO mice were infected intratracheally with S. aureus (5 X 10^7^ CFU/mouse). After 6h, the mice were euthanized and their BALF was collected, stained and subjected to flow cytometry. **(G)** Representative images of WT and ATF3 KO mice lung tissues were assessed for macrophage (F4/80) after S. aureus infection. **(H)** Representative dot plot showing CD11b+ F4/80 + cells. **(I)** Quantification of **(H)** Data are shown as the mean ± SD. *P < 0.05; ***P < 0.001 by Student’s *t* test.

Macrophages and neutrophils are necessary to control *S. aureus* infection in the lungs (18). Since macrophages and neutrophils are critical to the survival of pneumonia, we investigated whether the loss of ATF3 affects the recruitment of macrophages or neutrophils to the alveolar space during *S. aureus* pneumonia. ATF3 KO mice displayed profound lung pathology during the infection **(** **Figures 2A, B**). To further analyze the neutrophil accumulation in the lung parenchyma, we performed MPO assay and showed that the MPO activity was similar between the two genotypes (**Figure 2C**). We also found that WT and ATF3 KO mice had a comparable neutrophil number as reflected by s100a9 staining and Ly6G+/CD11b+ population in the FACS analysis in post-infection lung tissue sections (**Figures 2D, E**). In addition, the total number of infiltrating cells was very similar between the WT and ATF3 KO mice from BALF samples (**Figure 2F**). Interestingly, we noticed that, compared to WT mice, ATF3 KO mice had fewer macrophages recruited into alveolar spaces, as revealed by F/80 staining for the macrophages (**Figure 2G**). We further confirmed ATF3 controls F4/80 macrophage cell recruitment to the lung with histologic observations, the increased F4/80 macrophage cell in the WT mice lung through time-dependent manner during the S. aureus infection (added **Figure 1 Supplementary Data**). ATF3 KO mice also had a lower macrophage infiltration from BALF samples the FACS analysis and absolute cell counts (**Figures 2H, I**), which indicated that ATF3 had a host protective effect on survival through macrophage-mediated anti-*S. aureus* immunity. Collectively, in *S. aureus* infection, ATF3 exerts host protection by increasing the number of macrophages in the alveolar lavage fluid.

### ATF3 Enhances Macrophage Bacterial Clearance Ability

**Figure 3:**
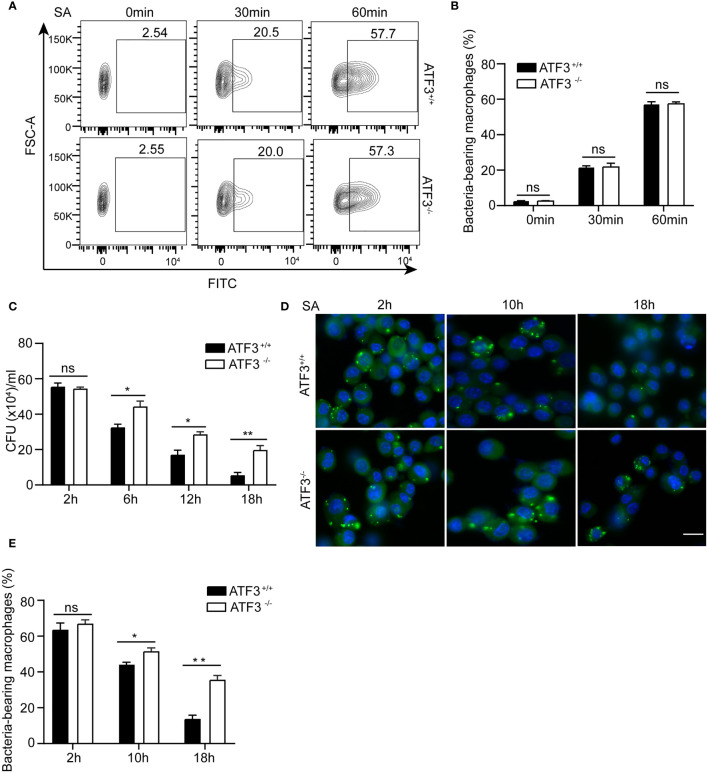
ATF3 enhances macrophage bacterial clearance ability. WT and ATF3 KO BMDM cells were infected with *S. aureus* (MOI = 1) for the indicated time periods. **(A, B)** The phagocytic ability was evaluated by taking up the percentage of macrophages with CFSE-labeled *S. aureus*. Representative plots and quantification by bar graphs from five independent experiments of flow cytometry are shown. **(C)** The bacterial load was measured and calculated in BMDM cells within the indicated time period after infection. **(D, E)** Intracellular bacteria measured by immunofluorescence microscopy. At 2, 10, and 18 h after infection, the uptake of *S. aureus* in BMDM cells is marked by CFSE (green). The nuclei are stained with DAPI (blue). Five representative images of each group were analyzed. Scale bar, 30 μm. ns, *P* > 0.05, *P < 0.05, **P < 0.01.

We next examined the effect of ATF3 on the ability of macrophages to clear bacteria. The phagocytic activity of macrophages was initially assessed by carboxyfluorescein succinimidyl ester (CFSE)-labeled *S. aureus*. There was no significant difference in the number of internalized bacteria between the WT and ATF3 KO macrophages (**Figures 3A, B**), indicating that ATF3 had no significant effect on macrophage phagocytosis. However, the count of bacterial colony-forming units (CFU) showed that the bacterial load was significantly reduced in WT, while ATF3 KO had less reduction during the 6-18 h infection process (**Figure 3C**). To further evaluate the bactericidal ability of macrophages, we conducted a lysostaphin protection test, in which the survival of internalized bacteria was observed with a fluorescence microscope (19). Remarkably, the number of living bacteria was increased in ATF3 KO macrophages compared to WT macrophages and lungs (**Figures 3D, E**), indicating that ATF3 enhanced the ability of macrophages to eliminate invading bacteria.

### ATF3 Enhances Macrophage Bacterial Clearance Independent of Lung Released Pro Inflammatory Cytokines

**Figure 4:**
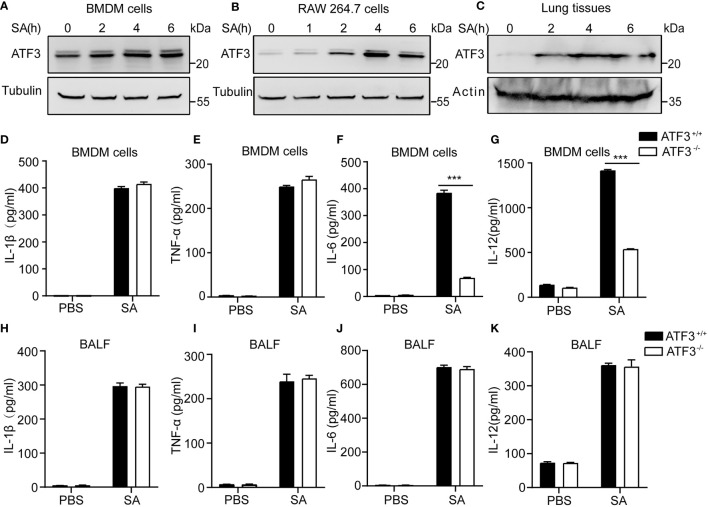
ATF3 enhances macrophage bacterial clearance independent of M1/M2 macrophages. WT and ATF3 KO mice were challenged with 5 × 10^6^ *S. aureus* colony forming units (CFU) and sacrificed 12 h later for subsequent functional analysis. **(A, B)** BMDM cells and RAW264.7 cells were infected with *S. aureus* for the indicated time periods. The cells were then lysed, and ATF3 protein levels were examined by immunoblotting. **(C)** Lungs obtained from *S. aureus*-infected mice for indicated time points were homogenized and ATF3 protein expression were measured by immunoblotting. **(D–G)** Macrophages derived from WT and ATF3 KO mice were infected with *S. aureus* for the indicated time periods, and cytokine levels in the cell supernatants were determined by ELISA. **(H-K)** WT and ATF3 KO mice were challenged with 5 × 10^6^ CFUs of *S. aureus* and sacrificed 6 h later for the subsequent functional analysis. The levels of BALF cytokines (IL-1β, TNF-α, IL-6, and IL-12) were detected by ELISA. ***P < 0.001.

Given the importance of macrophages in host defense against bacterial infections (5, 20), we next evaluated the effect of ATF3 on macrophages during staphylococcal infection. First, we noticed that ATF3 was induced in BMDM cells and RAW264.7 macrophages in a time-dependent manner after *S. aureus* infection (**Figures 4A, B**). Our previous study has reported that ATF3 is a crucial response gene in the lipid/cholesterol mediated inflammatory responses (14). The other study also showed that the transcriptional response suggests that the cholesterol *de novo* synthesis increases considerably in RAW264.7 cells to compare other macrophage cells, by those ATF3 was the top list of upregulated genes (21). It is indicated that ATF3 appears to have differing effects in immune response and is related to the metabolic regulation. Such an altered expression was also observed in the lungs from staphylococcal infection mice (**Figure 4C**). Indeed, the similar regulatory effect between WT and ATF3 KO significantly increased the expression of M1 proinflammatory cytokines, such as IL-1β, TNF-α (**Figures 4D, E**), with the exception of IL-6 and IL-12p40 being more significantly elevated in BMDM of WT (**Figures 4F, G**), while there were no differences in BALF among the major proinflammatory agents involved in the antimicrobial response *in vivo* (**Figures 4H**–**K**). In conclusion, our data suggest that ATF3 promotes the inflammatory response of macrophages in the early stages of staphylococcal infection regardness of lung released pro inflammatory cytokines.

### ATF3 Enhances Macrophage Bacterial Clearance Dependent on Antimicrobial Signature

**Figure 5:**
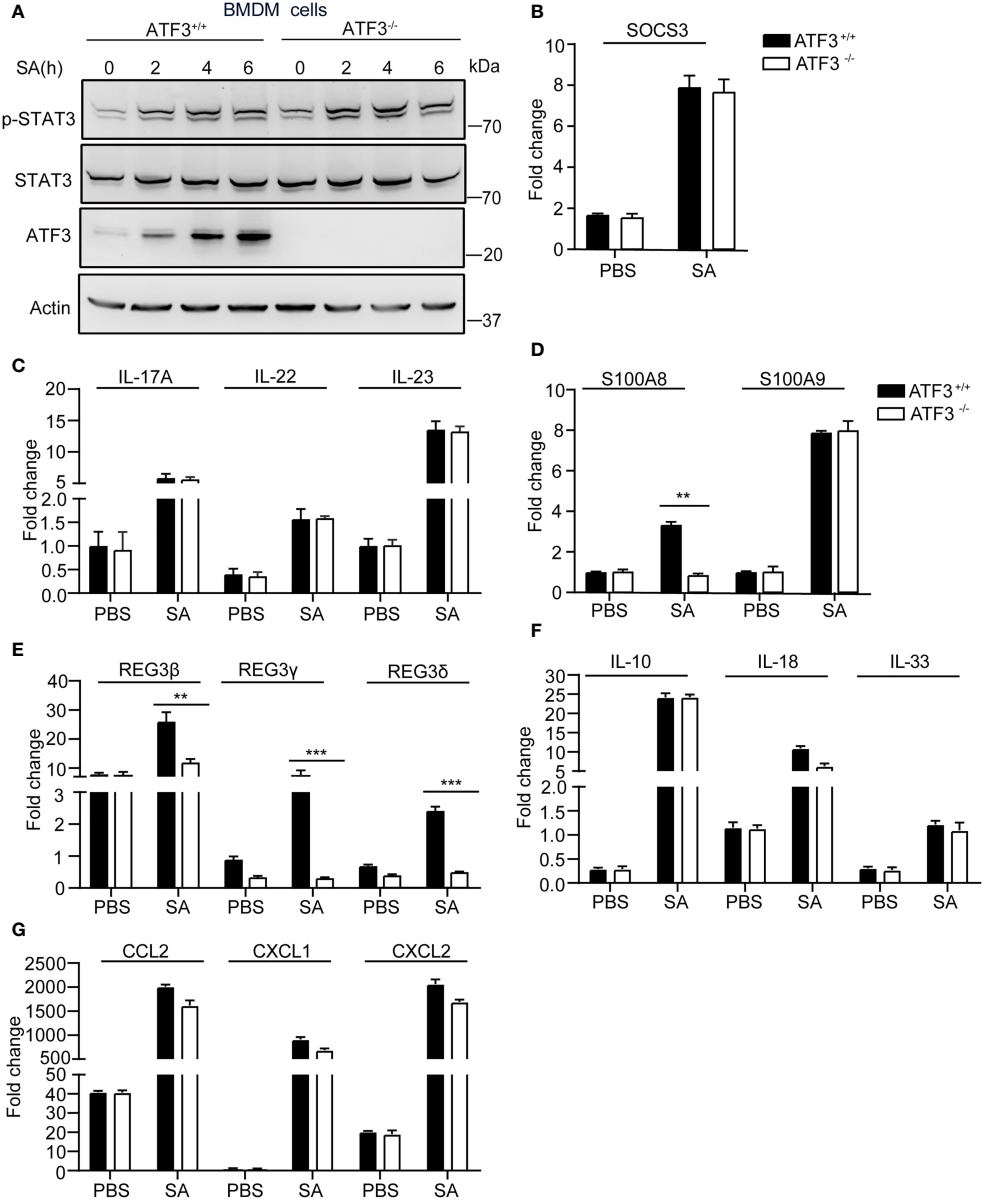
ATF3 enhances macrophage bacterial clearance dependent on an antimicrobial signature. WT and ATF3 KO BMDM cells or mice were infected with *S. aureus* (MOI = 1) for the indicated time periods. **(A, B)** The cells were lysed; the protein levels of STAT3 and p-STAT3 were analyzed, and the amount of SOCS3 mRNA was detected using quantitative PCR (qPCR). **(C–G)** qPCR analysis of the indicated cytokines (IL-17, IL-22, and IL-23), AMPs (S100A8,S100A9,REG3β, REG3γ, REG3δ), and other cytokines (IL-10, IL-18, and IL-33), chemokines (CCL2, CXCL1, and CXCL2) associated with bactericidal activity. All results are from three independent experiments and are expressed as mean ± SD. *P < 0.05, **P < 0.01, ***P < 0.001 by the Student’s *t* test.

There is increasing evidence that antimicrobial peptides (AMP) play a central role in restricting bacterial replication and preventing tissue damage (22). The AMP such as Regenerating islet-derived protein type 3 [Reg3] was regulated by the IL-17, IL-22/23 through stat3 signaling (23). First, we experimentally found no significant difference in STAT3 signaling between WT and ATF3 KO cells with respect to the expression of phosphorylated STAT3 and its target gene SOCS3 (**Figures 5A, B**). Given that IL-22 can mediate the expression of many AMPs, including Reg3 family and S100A8,9 (24), we examined whether ATF3 has an effect on AMP and analyzed the changes in IL-22 levels. Notably, increased levels of IL-22 and the related cytokines IL-17 and IL-23 were observed in macrophages infected by *S. aureus*, but no differences in induction of IL-17, IL-22, and IL-23 were observed between WT and ATF3 KO cells (**Figure 5C**). Interestingly, the expression levels of the molecules with antibacterial properties, such as S100A8 and Reg3 family genes were increased in WT macrophages but decreased in ATF3-null cells (**Figures 5D, E**). Further we found that WT and ATF3 KO BMDM cells had a comparable induced expression of cytokines, chemokines after S. aureus infection (**Figures 5F, G**). Collectively, our data suggest that ATF3 enhances the production of its antimicrobial effector molecules, thereby potentially enhancing the ability of macrophages to clear bacteria.

### ATF3 Directly Regulates the Antimicrobial Genes Through the ATF3 Binding Sites

**Figure 6:**
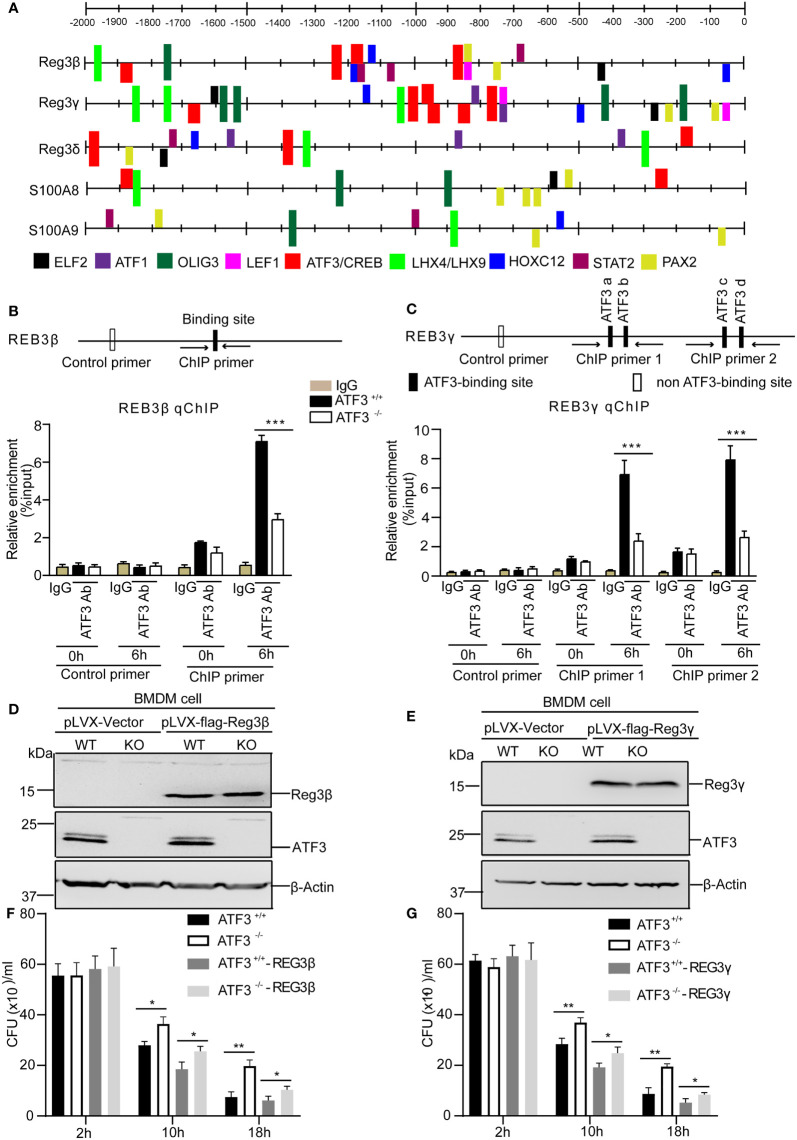
ATF3 directly regulates the antimicrobial genes through the ATF3-binding sites. **(A)** Schematic linear map shows the putative binding sites of ATF3 and other transcription factors of the AMP genes. **(B, C)** The diagram shows the regulatory regions of the AMP gene that contain or lack the high-affinity ATF3-binding site (black and white boxes on the map). ChIP assays was performed in WT and ATF3 KO cells using either anti-IgG or an anti-ATF3 antibody to assess binding ability at the putative ATF3 binding site in Reg3β and Reg3γ promoter pretreated with PBS or S. aureus. **(D, E)** The efficiency of WT and ATF3 KO BMDM cells stably over-expressing REG3β or REG3γ was assessed by immunoblotting. **(F, G)** WT and ATF3 KO BMDM cells and cells stably over-expressing REG3β or REG3γ were infected with *S. aureus* (MOI = 1) for the indicated time periods and then the bacterial load was measured and calculated in BMDM cells after infection. ns, *P* > 0.05, *P < 0.05, **P < 0.01, ***p < 0.001.

We next investigated the functional relevance of ATF3 induction by *S. aureus* infection with respect to AMP expression. We studied the regulatory role of the transcription factor ATF3 in AMP transcription. To explore whether ATF3 could regulate AMP expression, CiiiDER, a tool for predicting and analyzing transcription factor binding sites was used to analyze the potential ATF3 binding site in these AMP genes (25). In our computational prediction results, those AMP genes had at least one predicted ATF3-binding site (**Figure 6A**). Consistent with Reg3β and Reg3γ as a direct target, chromatin immunoprecipitation analysis revealed that endogenous ATF3 bound to different regions of Reg3β and Reg3γ regulatory elements containing ATF3 or ATF/CREB binding sites; such binding was abolished by ATF3 KO BMDM (**Figures 6B, C**). Hence, ATF3 seems to regulate the levels of Reg3β and Reg3γ *via* direct transcription control. To evaluate a role for antimicrobial protein Reg3, we overexpressed Reg3β and Reg3γ in macrophages. Immunoblotting of BMDM cell lysates showed the exogenous Reg3β and Reg3γ proteins (**Figures 6D, E**). Reg3β and Reg3γ have potent growth-inhibitory activity against S. aureus (**Figures 6F, G**). These data are consistent with the finding that Reg3 has selective bactericidal activity against pulmonary S. aureus infections (9).

### ATF3 Exerts its Effect on Macrophage Migration and Recruitment in Acute Lung Infection by Regulating F-Actin

**Figure 7:**
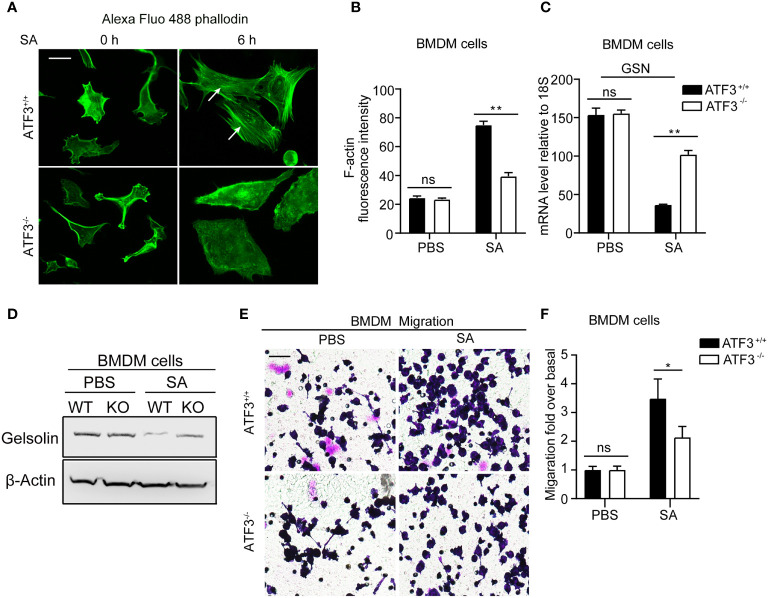
ATF3 regulates GSN and cytoskeleton remodeling for macrophage motility and recruitment. **(A)** Representative images of the actin cytoskeleton stained with phalloidin in WT and ATF3 KO BMDM cells and cells infected with *S. aureus* (MOI = 1). The white arrow indicates F-actin. **(B)** The intensity of F-actin is quantified on the right (*P < 0.05). **(C, D)** The GSN expression was analysis by the qPCR analysis and immunoblotting assay between WT and ATF3 KO BMDM cells with or without S. aureus infection. **(E)** The effect of S. aureus infection on WT and ATF3 KO BMDM cells migration was evaluated by trans-well assay in 24h. **(F)** Quantitative analysis of the cell migration ratio in **(E)**. ns, *P* > 0.05, *P < 0.05, **P < 0.01.

**Figure 2H** shows that ATF3 KO mice had a lower macrophage infiltration after *S. aureus* infection. To further investigate how ATF3 controls cell recruitment through motility, we studied the effect of ATF3 on the actin cytoskeleton. The polymerization and depolymerization of filamentous (F) actin have been reported to control the reorganization of the cytoskeleton, which is essential for cell movement through morphological changes (26). We next investigated whether ATF3 may regulate cellular recruitment by altering the actin cytoskeleton. We performed staining for F-actin in WT and ATF3 KO BMDM, revealing that there was no significant difference in actin stress fibers between WT and KO cells. Interestingly, in the case of S. aureus infection, we found that WT cells had significantly more actin stress fibers(**Figures 7A, B**).In addition, this impacted F-actin correlated with actin-modifying protein gelsolin (GSN) expression, which was regulated by ATF3, as described in our previous study (27). Positive or negative regulation of GSN by ATF3 is dependent on cofactors, such as different histone deacetylases (HDACs) in a context-dependent manner. Results showed that ATF3 negatively regulated the GSN mRNA and proteins expression in BMDM and GSN expression was higher in ATF3 KO cells compared to WT cells after S. aureus infection (**Figure 7C, D**). We further examined the role of ATF3 in cell motility and found no significant difference in the degree of migration of WT BMDM and ATF3 KO BMDM in the unstimulated conditions; however, WT BMDM were significantly more migratory than ATF3 KO cells in *S. aureus*-stimulated conditions (**Figures 7E, F**). These data show that ATF3 regulates cell motility *in vitro* and may drive macrophage recruitment in lung tissues.

## Discussion

Macrophages, like neutrophils, are important immune sentinels that contribute significantly to innate defense but are also involved in adaptive immunity. However, despite their ability to control microbial infections, they fail to eradicate *S. aureus*, leading to immune evasion and causing chronic persistent infections (28). Despite significant progress in understanding the molecular details of the interaction between *S. aureus* and macrophages, which play an important role in the clearance of bacteria, many questions remain to be answered. In this study, we showed that infection with *S. aureus* significantly increased ATF3 expression, which accelerated bacterial clearance and could reduce the symptoms of pneumonia in mice. Meanwhile, ATF3 KO mice were more susceptible to *S. aureus* infection, had a higher bacterial load, and experienced exacerbated pneumonia tissue damages. Importantly, we also found that ATF3 expression correlated with antimicrobial gene expression, which corresponded to bacterial killing. ATF3-deficient cells and lung tissues had reduced expression levels of antibacterial genes, which in turn resulted in altered antimicrobial capacity. Our further studies showed that the antimicrobial genes upregulated during the antimicrobial process were direct targets of the transcription factor ATF3 as confirmed by chromatin immunoprecipitation analysis. Furthermore, anti-*S. aureus* infection was primarily mediated by macrophages, which were regulated by ATF3-mediated chemokines and antimicrobial peptides to recruit macrophages to synergistically combat pathogens. The immune regulation of ATF3 against *S. aureus* infection primarily involved proinflammatory cytokines, but it was independent of the local release of cytokines, and also independent of STAT3 activation, leading to IL-17 and IL-22 expression.

One of the most important findings of this study is that ATF3 contributes to *S. aureus*-induced AMPs production. First, *S. aureus* infection induced the accumulation of ATF3, which in turn upregulated AMPs, and we also identified the increased AMPs genes containing the putative ATF3/CREB consensus pattern by ChIP analysis. Second, ATF3 regulated the expression of antimicrobial genes that correspond to bacterial killing, rather than those affecting phagocytosis. Third, ATF3 exerted a cell motility-dependent effect on macrophage recruitment by regulating the expression of the actin GSN.

The original study has reported that ATF3 functions to suppress TLR-mediated cytokine expression through a negative feedback loop. Subsequently, the function of ATF3 was applied to the study of multiple bacterial infection responses. During bacterial sepsis, hosts responded to infection by upregulating or inhibiting cytokines through ATF3 (29, 30). In the case of Gram-negative bacterial infection, ATF3 acted as a negative regulator to inhibit the production of inflammatory cytokines during the invasion of *Escherichia coli* and *Neisseria gonorrhoeae* (31, 32). Therefore, ATF3 KO mice showed longer survival times than WT controls after infection with Gram-negative bacteria due to the induction of ATF3-mediated sepsis-related immunosuppression on the major reactive oxygen species (ROS) condition (29). In contrast, in Gram-positive bacterial infections, ATF3 acted as a positive regulator to enhance the production of proinflammatory cytokines against pathogens such as *Streptococcus pneumoniae*, *Listeria monocytogenes*, and *S. aureus* (13). In the present study, we found that during *S. aureus* infection, ATF3 promoted the antimicrobial response, as well as macrophage infiltration, increasing the production of inflammatory cytokines. Proinflammatory cytokines, such as IL-6, TNF-α, and IL-1β, have been shown to play a guiding role in the production of antimicrobial cytokines or other effector molecules and contribute to resolving infections. For example, TNF-α induced by recruited monocytes is necessary for IL-17 production, macrophage phagocytosis, and bacterial clearance, and therefore, for facilitating recovery from pneumonia (33). To date, *S. aureus* remains one of the leading causes of iatrogenic and community-associated infections, with high mortality and with limited therapeutic options, while the functional significance of ATF3 in Gram-positive infections remains poorly understood. ATF3 KO mice are sometimes more susceptible and sometimes more resistant to bacterial infections, regardless of Gram-negative or positive strain or the action of inflammatory cytokines. It is more dependent on the antimicrobial response *via* AMPs.

It has been reported that IL-17 and IL-22 can induce the expression of AMPs that can kill or inactivate microorganisms by regenerated islet-derived protein 3 (Reg3) and defensins (34). Accordingly, the loss of IL-17 or IL-22 leads to a higher lung bacterial load and severe staphylococcal pneumonia (35). The production of IL-17 and IL-22 is regulated by a key signal event from STAT3 activation (36). In our study, the loss of ATF3 did not alter the STAT3 phosphorylation; subsequently, we also analyzed the downstream target genes of STAT3, such as IL-17 and IL-22, or AMPs; we only found a decrease in Reg3 and S100A8, but no difference in IL-17 and IL-22 between WT and ATF3 KO BMDM after *S. aureus* infection. These data indicated that ATF3 may be downstream of STAT3 and IL-17/IL-22 signaling, and some studies have indicated Toll-like receptor (TLR)-induced ATF3 by c-Src (30), which may pass STAT3 or crosstalk to STAT3 downstream signaling, such as AMPs genes. Whether those kinases can regulate ATF3-mediated cellular antimicrobial and migration needs further characterization. Our current study elucidated a regulatory mechanism mediated by the ATF3/AMPs axis, which plays a key role in modulating macrophage antibacterial responses. However, since ATF3-driven antimicrobial signaling is also triggered by other immune cell subpopulations, such as innate lymphocytes and T cells (37), we think that the early response and macrophage-mediated antibacterial infection may exclude the effect of other immune cells’ response. In addition, Alveolar macrophages (AMs) are a lung-specific type of Tissue-resident macrophages (TRM). They play a central role in maintaining alveolar homeostasis by removing cellular debris, excess surfactant, and inhaled bacteria. Still, they are also crucial in preserving lung function during pulmonary infections. Two alveolar epithelial cell types surround AMs. alveolar type 2 cells (AT2s) produce surfactant, act as facultative progenitors in case of alveolar injury, and activate the immune system in response to pathogen-related stimuli. Granulocyte-macrophage colony-stimulating factor (CSF), also known as CSF2, and granulocyte CSF, also known as CSF3, are important survival and proliferation factors for neutrophils and macrophages. AT2-derived GM-CSF continues to be a critical niche factor for the maintenance of AM in the adult alveoli (38). In our case, CSF2 and CSF3 were significantly induced by S. aureus infection at WT lung, which may support long term macrophages survival, but not for short term (few hours) macrophages migration (added **Figure 2 Supplementary Data**). ATF3 mediated macrophages recruitment finding was supported by the mechanical of ATF3 regulates the actin cytoskeleton of F4/80 macrophages within the cell for their migration, rather than cytokines and chemokine mediated the cell recruitment.

Our data indicate that ATF3 plays a role in repressing the ability of GSN to sever actin stress filaments (depolymerization) and trigger macrophage migration consistent with the recent report that lipopolysaccharide (LPS) induced the expression of microRNA miR-21, which downregulated GSN expression and reversed the high-density phenotype, indicating the high motility of macrophages (39). Epigenetic repression of the tumor suppressor GSN is frequently observed in cancers, and chronic inflammation can promote tumor progression *via* aberrant DNA methylation (40). Downregulated GSN leads to increased cell mobility through actin stress filaments, which can promote cancer metastasis (27). We propose here that the low level of GSN in macrophages may be due to the increased expression of ATF3, and that ATF3 is directly bound to GSN regulatory elements. Other reports have indicated that epigenetic silencing plays a role in the regulation of GSN, and histone deacetylase (HDAC) and DNA methylation inhibitors both increase GSN levels. Others have previously reported that ATF3 interacts with HDAC1 (31), which may be one of the reasons why ATF3 negatively regulates GSN. Our current findings that *S. aureus* induces ATF3 and regulates GSN-mediated F-actin polymerization fill a gap in the molecular regulatory mechanism of macrophage motility function, which is consistent with recent reports that TLR2 or LPS induces macrophages by enhancing actin polymerization and cell migration (41, 42).

In conclusion, this study revealed that the role of ATF3 in innate immunity involves the regulation of macrophage recruitment and function during early *S. aureus* infection. Furthermore, ATF3 contributes to bacterial killing in both lung tissues and BMDM cells, which is related to the regulation of AMPs gene promoter and their expression. ATF3 also triggers filamentous (F-actin) to control cytoskeletal reorganization in macrophages, thereby leading to morphological changes critical to cell motility in the intra infection sites of the lung, which may lead to antimicrobial processes within the cells.

## Data Availability Statement

The raw data supporting the conclusions of this article will be made available by the authors, without undue reservation.

## Ethics Statement

The animal study was reviewed and approved by Ruijin Hospital, Shanghai Jiao Tong University School of Medicine.

## Author Contributions

DX contributed to the design and implementation of the research. YD performed most of the *in vitro* and *in vivo* experiments. ZM performed *in vivo* experiments. JZ, SH, and YS performed *in vitro* experiments. XY performed data acquisition and analysis. YD and DX drafted the article. LS supervised the study and provided critical reagents. All authors contributed to the article and approved the submitted version.

## Funding

This work was supported by project grants from the National Natural Science Foundation of China (NSFC) (81871274, 82071811, and 31670905).

## Conflict of Interest

The authors declare that the research was conducted in the absence of any commercial or financial relationships that could be construed as a potential conflict of interest.

## Publisher’s Note

All claims expressed in this article are solely those of the authors and do not necessarily represent those of their affiliated organizations, or those of the publisher, the editors and the reviewers. Any product that may be evaluated in this article, or claim that may be made by its manufacturer, is not guaranteed or endorsed by the publisher.
