# Supplementary material for: ATF3 Positively Regulates Antibacterial Immunity by Modulating Macrophage Killing and Migration Functions
Source: Front Immunol. 2022 Mar 16;13:839502. doi: 10.3389/fimmu.2022.839502 (PMC8965742; doi:10.3389/fimmu.2022.839502)
Supplement: Supplementary file 1 [file DataSheet_1.pdf]

SUPPLEMENTARY FIGURES AND TABLE

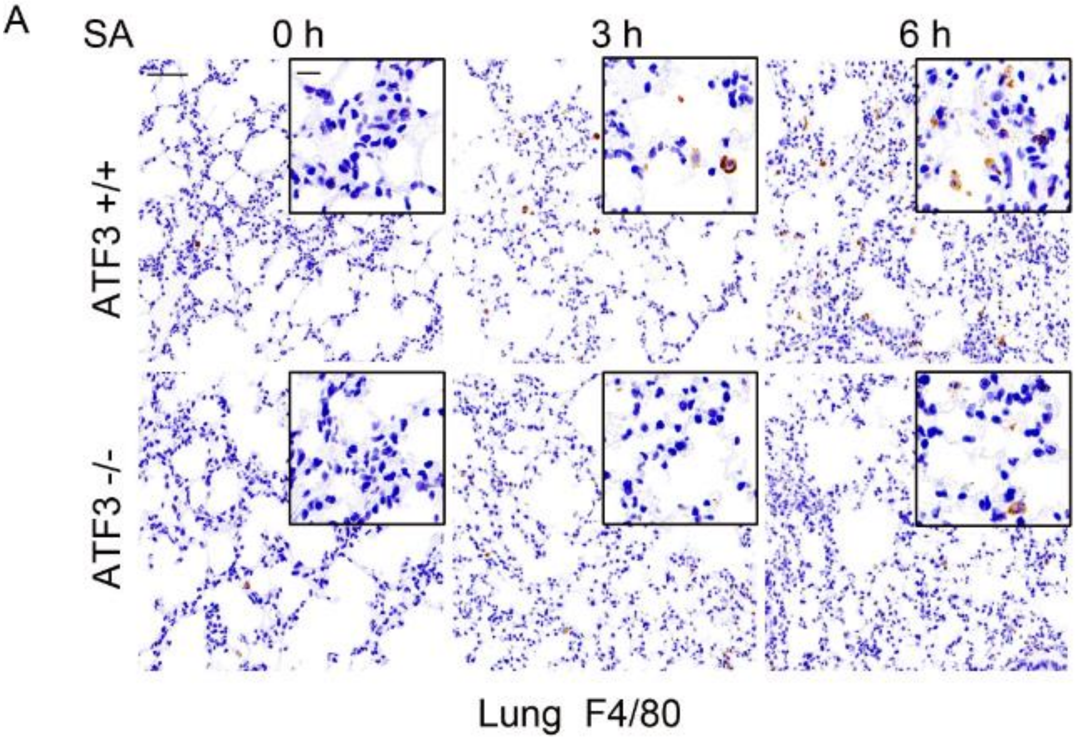

Supplementary Fig. 1
(A) Representative images of WT and ATF3 KO mice lung tissues were assessed for macrophage (F4/80) after S. aureus infection for 0,3, or 6 h.

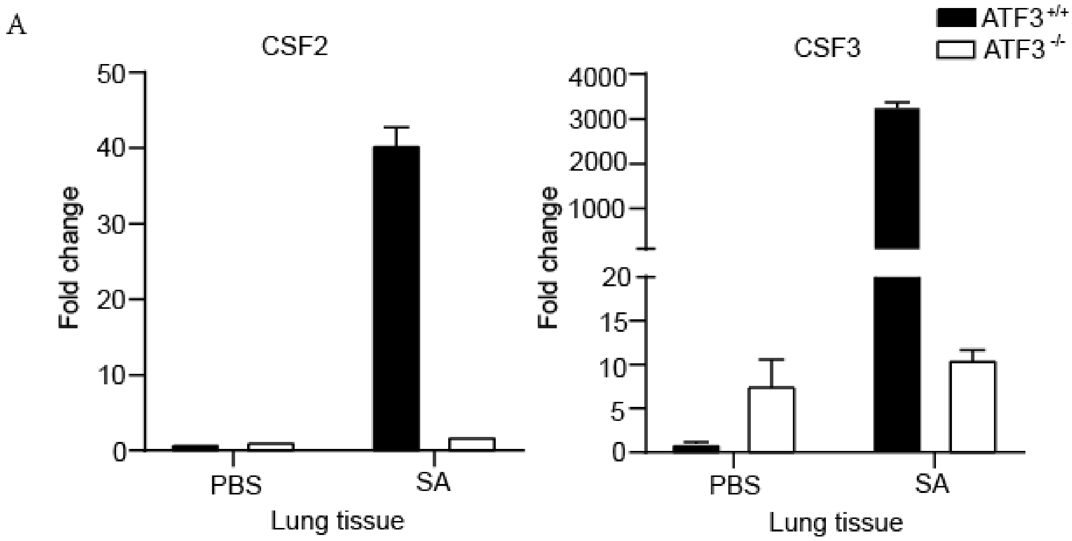

Supplementary Fig. 2
(A) WT and ATF3 KO mice were infected with *S. aureus* (5×10^7 CFU/mouse) and then euthanized at 6 h and the expression level of lung factors such as CSF2 and CSF3 in lung tissue were detected using quantitative PCR (qPCR).

Supplementary Fig. 3 | Uncropped western blot images of the indicated Figures.

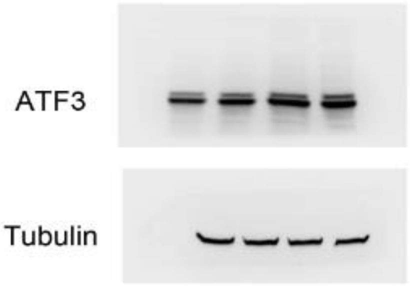

Figure 4A

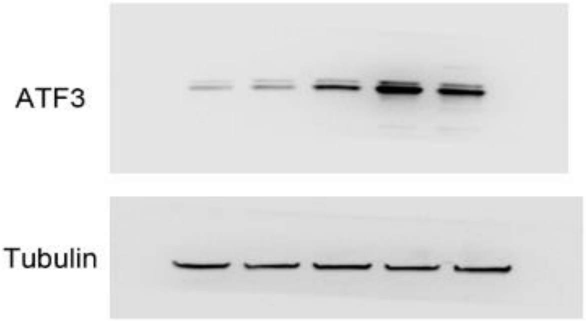

Figure 4B

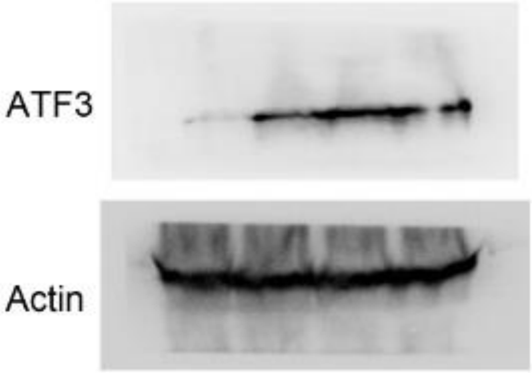

Figure 4C

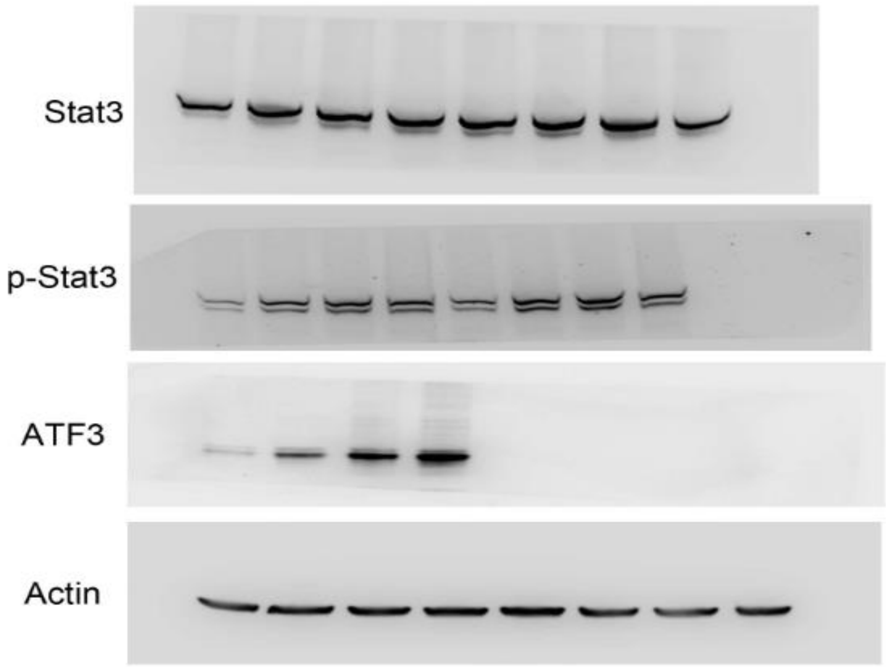

Figure 5A

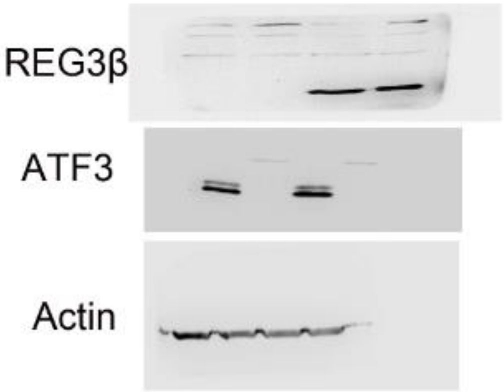

Figure 6D

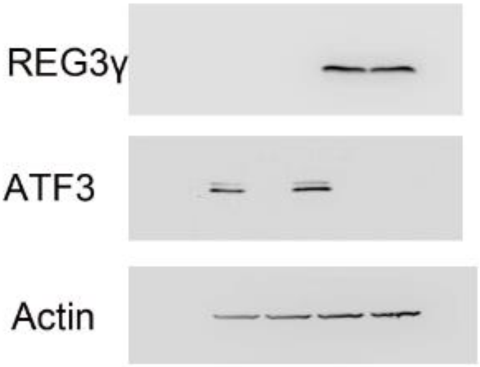

Figure6E

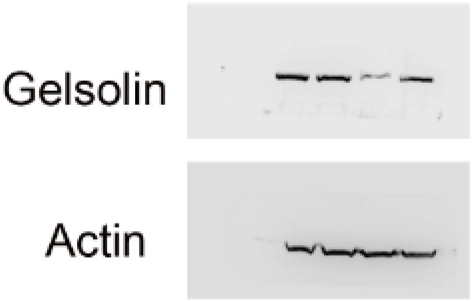

Figure7D

Supplementary Table 1. Primers used for qPCR

Name	Primer sequence (5’-3’)
Socs3-F	GGACCAAGAACCTACGCATCCA
Socs3-R	CACCAGCTTGAGTACACAGTCG
CXCL1-F	CGCTTCTCTGTGCAGCGCTGCT
CXCL1-R	CAAGCCTCGCGACCATTCTTGA
CXCL2-F	TCCAGAGCTTGAGTGTGACG
CXCL2-R	TCCAGGTCAGTTAGCCTTGC
S100A8-F	TGTCCTCAGTTTGTGCAGAATATAAA
S100A8-R	TCACCATCGCAAGGAACTCC
S100a9-F	AAAGGCTGTGGGAAGTAATTAAGAG
S100a9-R	GCCATTGAGTAAGCCATTCCC
Il10-F	ATGCTGCCTGCTCTTACTGACTG
Il10-R	CCCAAGTAACCCTTAAAGTCCTGC
IL18-F	ACCTCCAGCATCAGGACAAAG
IL18-R	TGTACAGTGAAGTCGGCCAAAG
IL-33-F	TCCAACTCCAAGATTTCCCCG
IL-33-R	CATGCAGTAGACATGGCAGAA
Ccl2-F	TTAAAAACCTGGATCGGAACCAA
Ccl2-R	GCATTAGCTTCAGATTTACGGGT
Reg3α-F	GGCACCGAGCCCAATG
Reg3α-R	GGATTTCTCTCCCATGCAAAGT
Reg3β-F	ATGGCTCCTACTGCTATGCC
Reg3β-R	GTGTCCTCCAGGCCTCTTT
Reg3γ-F	CGTGCCTATGGCTCCTATTGCT
Reg3γ-R	TTCAGCGCCACTGAGCACAGAC
Reg3δ-F	ACCACAGACCTGGGCTAATG
Reg3δ-R	AGTCCAATCCAGATGTATGGGAA
IL17a-F	TTTAACTCCCTTGGCGCAAAA
IL17a-R	CTTTCCCTCCGCATTGACAC
IL-22-F	ATGAGTTTTTCCCTTATGGGGAC
IL-22-R	GCTGGAAGTTGGACACCTCAA
IL-23-F	CAGCAGCTCTCTCGGAAT
IL-23-R	ACAACCATCTTCACACTGGATACG
Gelsolin-F	CAAAGTCGGGTGTCTGAGGC
Gelsolin-R	CAGGCACCAGGTCAAACTTCTCC
18S rRNA-F	GGCTGTATTCCCCTCCATCG
18S rRNA-R	CCAGTTGGTAACAATGCCATGT
β-actin-F	CTCATGAAGATCCTGACCGAG
β-actin-R	AGTCTAGAGCAACATAGCACAG

CSF2-F	GGCCTTGGAAGCATGTAGAGG
CSF2-R	GGAGAACTCGTTAGAGACGACTT
CSF3-F	CTCAACTTTCTGCCCAGAGG
CSF3-R	TAGGTGGCACACAACTGCTC

Supplementary Table 2. Primer pairs used for ChIP

Primer pairs	Sequence
Promo-Reg3β -forward	GTTAACCCCATATACTCCTAGG
Promo-Reg3β -reverse	ATAATCATGCTACAATCCACAG
Reg3β-Cont- forward	CAGGAACACCTTGAGCATCAATT
Reg3β-Cont- reverse	AAGTGGCCTCCTCCTATAGCCA
Promo-Reg3γ-forward	TAACCTCATGAACACAAGTGTTG
Promo-Reg3γ-reverse	ATTCTGAATTGTTTACTTGATAT
Promo-Reg3γ-forward	TCAAATCATTTTAGCCTTGCT
Promo-Reg3γ-reverse	TTCACGTTTCACATTAGAATG
Reg3γ-Cont- forward	GTATATTACATAGTGGGATTCA
Reg3γ- Cont- reverse	AAGATTTCTAAGAGGAAAGTAAT
